# Multi-Agent Reinforcement Learning for Traffic Flow Management of Autonomous Vehicles

**DOI:** 10.3390/s23052373

**Published:** 2023-02-21

**Authors:** Anum Mushtaq, Irfan Ul Haq, Muhammad Azeem Sarwar, Asifullah Khan, Wajeeha Khalil, Muhammad Abid Mughal

**Affiliations:** 1 Pakistan Institute of Engineering and Applied Sciences, Islamabad 44000, Pakistan; 2 PIEAS Artificial Intelligence Center (PAIC), Islamabad 44000, Pakistan; 3 Department of CS and IT, University of Engineering and Technology, Peshawar 25000, Pakistan

**Keywords:** Intelligent Transportation Systems, traffic flow management, deep reinforcement learning, autonomous vehicles, Multi-Agent Reinforcement Learning, multi-intersection signal control

## Abstract

Intelligent traffic management systems have become one of the main applications of Intelligent Transportation Systems (ITS). There is a growing interest in Reinforcement Learning (RL) based control methods in ITS applications such as autonomous driving and traffic management solutions. Deep learning helps in approximating substantially complex nonlinear functions from complicated data sets and tackling complex control issues. In this paper, we propose an approach based on Multi-Agent Reinforcement Learning (MARL) and smart routing to improve the flow of autonomous vehicles on road networks. We evaluate Multi-Agent Advantage Actor-Critic (MA2C) and Independent Advantage Actor-Critical (IA2C), recently suggested Multi-Agent Reinforcement Learning techniques with smart routing for traffic signal optimization to determine its potential. We investigate the framework offered by non-Markov decision processes, enabling a more in-depth understanding of the algorithms. We conduct a critical analysis to observe the robustness and effectiveness of the method. The method’s efficacy and reliability are demonstrated by simulations using SUMO, a software modeling tool for traffic simulations. We used a road network that contains seven intersections. Our findings show that MA2C, when trained on pseudo-random vehicle flows, is a viable methodology that outperforms competing techniques.

## 1. Introduction

Conventional traffic management strategies have proved inefficient as vehicular traffic continues to grow. Urbanization has led to a significant increase in traffic congestion and accidents in metropolitan areas. To accommodate the ever-increasing demands on transportation networks today, an Intelligent Transportation System is required. Congestion tends to prolong, impede, and be counter-intuitive to a wide range of economic activity in the city. The studies on traffic-related concerns and hazards are conducted in a variety of disciplines, including the environment and public health [1]. Communication and robotics breakthroughs have significantly impacted our daily lives and transportation. The sustainable development of Autonomous Vehicles technology targets lowering the number of traffic accidents, the amount of fuel consumed, emissions, and vehicular congestion through certain technological advancements. However, the prohibitively high costs have always hindered the large-scale production of driverless vehicles [2,3]. Globally, the rapid advancement of communications technologies has made AVs a crucial business paradigm. In response to emerging new and innovative ideas such as social media platforms, smartphones, and autonomous vehicles, some experts stated unequivocally that the transportation landscape is rapidly changing [4,5,6].

An autonomous vehicle can operate independently of human control and does not require manual interaction. Modern AVs can sense their immediate environment, classify the various types of objects they encounter, and interpret sensory data to determine the most appropriate navigation paths while adhering to traffic laws. AVs and smart routing also hold great potential for the improvement of road safety and significantly minimizing the number of accidents. Nowadays, many active safety technologies such as smart tires [7], intelligent signals [8], and smart signs [9] are helping vehicles to take control from human drivers. Significant progress has been achieved in responding appropriately to unexpected events, such as a backlash in the vehicle systems or a medium in the external environment that does not react as predicted by internal prototypes such as congestion [10]. However, it is vital to combine multiple techniques and develop new methodologies for successful autonomous navigation in these settings, as AVs can exacerbate the traffic situation if not managed appropriately.

Traffic signal management is an essential method for the management of road intersections and regulating urban road traffic. The inefficiency of this system can cause serious problems such as long travel delays, congested queues, and energy wastage, and in some situations, it might be a reason for accidents [11,12]. Optimizing traffic signals is crucial because it contributes to higher traffic throughput, possibly lower congestion, and more efficient traffic networks. Simultaneously, efforts to improve traffic control could be challenging because, realistically, the traffic system is taken as a complex nonlinear system with large state-action spaces. The sub-optimal control actions can easily result in congestion that eventually spread and thus are difficult to break down [13]. Current traffic signal controllers are embedded with fixed programs ignoring real-time conditions of traffic or considering it to a very limited degree [14]. The pre-time fixed traffic signals use an equal interval of time duration for each cycle or it can also use historical data for different duration of time. In practice, most traffic lights employ straightforward protocols such as switching between the traffic light modes (red and green) alternatively at predetermined intervals. The duration of these predetermined intervals may change due to some specific conditions, i.e., peak hours but is not optimized in any other way. Due to the inefficiency of such controllers, several researchers have explored the use of machine learning and artificial intelligence methodologies to develop efficient control algorithms.

Reinforcement learning is all about improving the numerical performance metric toward achieving a long-term goal by learning how to control a system in multiple ways. Receiving feedback on the actions or predictions made by the learning agent is what delineates reinforcement learning from the supervised machine learning methods. Moreover, the future state of a completely controlled system is most likely to get influenced by the predictions made and might affect the system in a long run. As a result, time plays a significant part. The main goal of reinforcement learning over a controlled system or environment is to formulate practical machine learning algorithms and comprehend their advantages and disadvantages. Due to its several practical applications, Reinforcement Learning has gathered considerable interest in performing Artificial Intelligence (AI) based control engineering or operations research [15]. Reinforcement Learning could be implemented on traffic signals in two ways, One is Single Agent Reinforcement Learning (SARL), and the other approach is Multi-Agent Reinforcement Learning (MARL). In SARL environment is explored and actions are performed by a single agent while in MARL more than one agent or group of autonomous, interacting agents collaboratively perform actions to explore the environment. SARL is very successful in many fields but there are certain limitations associated with SARL algorithms when it comes to many real-world problems that cannot be solved by a single active agent. MARL overcomes this challenge by working with several agents concurrently and learning how to solve specific problems by interacting with each other and with the environment [16]. The implementation of many large and successful applications involves MARL along with the deep neural network as function approximation [17]. The multiple autonomous agents in the MARL environment operate sequentially in decision-making problems. The aim of each agent is to optimize its reward by interacting with each other and the environment. MARL algorithms can be used in many real-world problems such as traffic control, robot systems, analysis of social dilemmas, energy distribution, and network packet routing because the partial observability in MARL is far more common than in SARL. Multi-Agent systems help to solve many challenges that arise in pre-programmed behaviors of agents and give a natural way to perceive a system or give an alternate way to perceive a system that is originally considered as centralized [18]. The multiple agents in the MARL system collaborate and exchange information. This experience sharing makes Reinforcement Learning (RL) system learn faster and improve performance with similar tasks [19]. The MARL agents that already gain some experience can help other learner agents or learner agents can imitate them for experience to speed up the learning process [20]. This results in efficient traffic signals that take immediate decisions by considering the current state of the roads.

Currently, the management of busy or major road intersections is primarily accomplished through traffic signals, whose inefficient operation results in a slew of problems, including lengthy delays for travelers and massive energy waste. Worse yet, it may result in vehicular accidents. Conventional traffic signal control systems either use fixed schemes that ignore real-time traffic or take traffic into account to a very limited extent [21]. The pre-timed fixed traffic signals set the duration of traffic signals equal in each cycle or different based on historical data. Particular control systems receive inputs from sensors such as loop detectors to determine the presence of vehicles at the intersection. However, the inputs are very densely processed to ascertain the duration of green/red lights. To address the above mention challenges, here are the contributions of this paper.

- We formulate the traffic signal control problem as Markov Decision Process (MDP) and apply MARL algorithms (IA2C & MA2C) to obtain dynamic and efficient traffic signal control Policies. The deep reinforcement learning-based framework for traffic signal control makes traffic lights intelligent enough to take decisions dynamically by observing the current state of the intersection.
- We used a rerouting technique for the vehicles coming behind the intersection by assigning them alternate paths to reduce the congestion on the road network and to manage the flow of traffic.

All the simulations are done in the simulation tool SUMO. The results show the effectiveness of our proposed approach to reduce congestion, waiting time, delay, and long queue lengths. The rest of the paper is organized as follows: Section 2 represents related work in the field. Section 3 represents the proposed methodology while Section 4 shows the experimental setup. Section 5 represents the results and discussion and Section 6 concludes the paper.

## 2. Related Work

A complex stochastic process determines traffic in real-time. The main challenge to address while considering optimizations in traffic flow with real-time traffic is to control green traffic light signal interval duration at the intersection. There is a substantial amount of research designed to decrease road user delay using MARL approaches [22]. Presents an effective approach to solving a traffic signal control problem using a Multi-Agent control system. It covers the various traffic optimization techniques used and demonstrates that the signal control problem is a suitable testbed for the MARL strategy. The authors in [23] use a framework of general-sum stochastic games to study MARL. Under this paradigm, they develop a Multi-Agent Q-learning approach and demonstrate that it converges to a Nash equilibrium under defined circumstances. This technique is advantageous for determining the optimum strategy when the game has a singular Nash equilibrium. When a game has several Nash equilibria, this approach should be used with other learning methods to determine optimum solutions. Similarly, Multi-Agent Reinforcement is used in many domains, such as in [24] the authors assert in this work that during training the policies of RL agents are at high risk of getting over-fit due to the several policies learned by independent RL, resulting in vague generalization during execution. The authors offer a new measure, joint-policy correlation along with a new deep reinforcement learning technique to quantify this impact and provide a generic approach based on Multi-Agent Reinforcement Learning for approximating optimal answers to mixtures of policies created and to select policies based on meta-strategies.

The authors in [25] explore the challenge of many agents perceiving and behaving in their environments to maximize their collective benefit. Agents must learn communication protocols in these settings to communicate the information expected to accomplish jobs. They used deep neural networks to demonstrate end-to-end learning of protocols in complicated contexts. this method helped them to counter Multi-Agent computer vision issues with partial observability.

A considerable portion of the research on Multi-Agent systems addresses different reinforcement learning approaches. The article [26] presents an in-depth examination of MARL approaches. The MARL algorithms reported in the literature are either explicitly or implicitly aimed at one of these two objectives or a mix of the two in a completely competitive, cooperative, or more generic environment. This article discusses a representative selection of these algorithms in-depth, along with the unique difficulties in each category. Additionally, the merits and drawbacks of MARL are discussed, and many application fields for MARL approaches.

Optimal regulation of traffic lights at intersections, or traffic signal management, is critical for decreasing the average amount of delay encountered, especially in light of the fast growth in vehicle usage. The [27] formulates the traffic signal problem as a discounted cost Markov decision process (MDP), and dynamic traffic signal policies are obtained using Multi-Agent Reinforcement Learning (MARL) methods. Each signaled intersection is modeled as an individual agent. using Round-Robin Reinforcement Learning or DQNs, the signal length of an agent’s phases is determined. Deep Q-Nets or DQNs utilize either greedy or Upper Confidence Bound (UCB) based exploration techniques. The Q factors are adjusted depending on the cost feedback collected from nearby agents. This made creating a feedback signal easy and has been demonstrated to reduce the average amount of delay of the autonomous vehicles present in the network. They also demonstrated using VISSIM simulations, that their methods outperform conventional methods. Finding the best scheduling technique for a traffic signal is challenging when dealing with traffic signal control for a large-scale traffic area. MARL is a potential technique for addressing this issue. The paper [28] proposes a novel MARL termed cooperative double Q-learning (Co-DQL), which has numerous distinguishing characteristics. Co-DQL is also based on a Q-Learning approach, instead, it is highly scalable as it implements an independent double Q-learning technique. this double Q-learning technique utilizes Upper Confidence Bound (UCB) and double estimators to design a strategy capable enough to eliminate the overestimation problem associated with standard Q-learning techniques with exploration. It models agent interaction using mean-field approximation, therefore assisting agents in developing a more cooperative approach. To enhance the learning process’s stability and robustness, authors develop a novel reward distribution mechanism and a technique for exchanging local states. Additionally, they examine the suggested algorithm’s convergence qualities. Co-DQL is applied to TSC and validated using TSC simulators under different traffic flow situations.

Adapting MARL methods to the traffic signal problem involves a few issues, as agents generally react to environmental fluctuations individually, but their collective behavior may not be optimum. The authors in [29] present the integration of a unique MARL system designed specifically for controlling traffic in an adaptive traffic signal control-based integrated environment, MARLIN-ATSC. MARLIN-ATSC can either be used in two different modes. Mode 1: an independent model in which each agent is considered an independent entity and is controlled accordingly. Mode 2: Integrated mode, which controls signals based on communication between surrounding junctions. MARLIN-ATSC is evaluated during the morning rush hour, the simulated road network consists of 59 intersections in the lower downtown area of Toronto, Canada. The collected results demonstrate that on average, the amount of average delay at each intersection has been reduced by a huge factor of 27% as in mode 1 to a distinguishing factor of 39% in mode 2. The amount of reduction in the average delay saves travel time of vehicles up to 15% in mode 1 and 26% in mode 2 along Downtown’s main routes.

Early approaches for traffic management either use single-agent RL methods or only use Multi-Agent algorithms to reduce the delay of vehicles at intersections. They are not considering the congestion other than the intersection. In this paper, we are proposing a Multi-Agent RL framework for traffic management at intersections and routing of vehicles to alternate paths to manage the flow of traffic other than the intersections. In a Multi-Agent environment, the experience sharing between agents improves their performance by making them learn faster similar tasks. The rerouting of traffic behind the intersections to the most favorable routes by the coordination of signals further improves performance. In our previous related work [30], we proposed a Q-learning-based RL framework using a single agent for the intersection and routing of vehicles. This presented paper is an extension of work by using Multi-Agent algorithms with rerouting, using almost seven intersections, and managing bigger road networks. In another of our earlier related work [31] we used platooning and collision avoidance strategies to improve the flow of traffic. In the current study, we are using large road networks, more intersections, more vehicles, and more complex algorithms to make the traffic flow system more efficient and reliable.

## 3. Proposed Methodology

**Figure 1:**
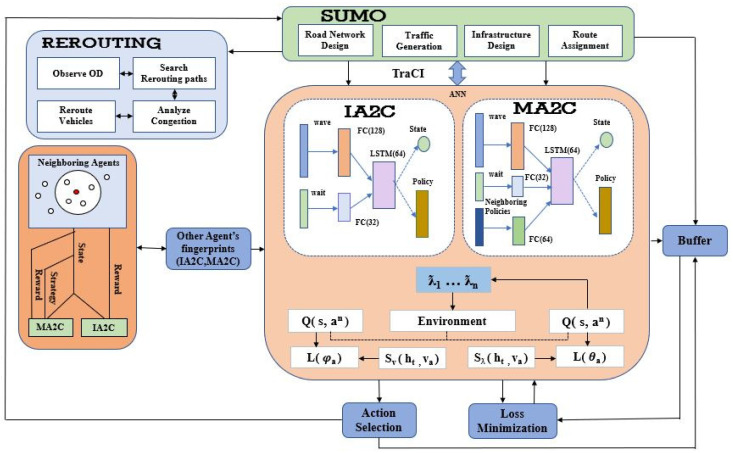
Proposed Approach: Traffic Flow Management using Independent Advantage Actor-Critic (IA2C), Multi-Agent Advantage Actor-Critic (MA2C) and Routing of vehicles.

In this paper, we used Multi-Agent Reinforcement Learning to control traffic signals and optimize traffic flow. We represent real-time traffic as a complicated stochastic process. We optimized the duration of the green and red phases in an intersection to enhance traffic flow in real time. MARL uses the Markov decision process (MDP) that provides an excellent framework for accomplishing this goal. There exists a direct proportionality between state size, action space, and the number of junctions present in a road network. As the total number of junctions in the road network increases, the state size and action space also increase exponentially. As a result, Markov Decision Process (MDP) are be required for the solution. Multi-Agent Reinforcement Learning (MARL) offers a systematic mathematical framework for addressing this issue. RL techniques are well-suited for this task since they are asynchronous in design and can learn efficient environment control strategies through experience. Systems implementing Multi-Agent Reinforcement Learning are highly effective since each agent views and controls a subset of the state space helping to reduce the state size and action space. In this paper, we proposed an approach for the congestion management of autonomous vehicles to improve the flow of traffic using MARL and to route vehicles to different routes. One of the critical challenges of MARL is managing large networks where each agent is behaving independently and has a limited amount of information about the entire network configuration. We have to use decentralized policies for such systems by considering network structures. Following are the methodological steps we followed in this work as shown in Figure 1:

- In the first step, We are using the SUMO traffic simulator for the generation of traffic, Road Network Design, Infrastructure Design, Route Assignment, traffic signals, and other related tasks.
- In the second step, We are implementing Independent Advantage Actor-Critic (IA2C) algorithm and training the Neural Network Model. The traffic signal switching is determined by observing the flow of nearby vehicles.
- In the third step, We are implementing Multi-Agent Advantage Actor-Critic (MA2C) algorithm and training the Neural Network Model. Here the traffic signal switching is also determined by observing the flow of nearby vehicles but the difference is that it also interacts with neighboring agents.
- In the fourth step, we apply the rerouting technique on both algorithms and improve the flow of traffic more effectively.

We are using mean-field type agents that are homogeneous and execute MARL using centralized training for decentralized execution. Centralized training for decentralized execution means the training of agents is done offline based on centralized information, but the execution is done decentralized. During training, all agents collect local information about their neighboring states, and after the completion of the training process, this information is used for decentralized policies where only data from the current state is required. The actor-critic approach with over-parameterized neural networks is used for developing efficient reinforcement learning algorithms. The objective of RL is to maximize the reward under the MDP framework. The agent observes the state $x_{t}\in X$ and performs action $y_{t}\in Y$ and observes policy π(x|y). An immediate reward $z_{t}=z\left(x_{t},y_{t},x_{t+1}\right)$ is received when the transition from the current state to the next state is made as $x_{t+1}∼P\left(\cdot |x_{t},y_{t}\right)$. The sum of immediate rewards that is total final reward under policy π is calculated as $Z_{t}^{\pi }=\sum_{t=1}^{\infty }d^{z-1}z_{t}$ where *d* is the discount factor. The following Q-function is used to calculate the expected total return: $Q^{\pi }\left(x,y\right)=E\left[Z_{t}^{\pi }x_{t}=x,y_{t}=y\right]$ and the optimal Q-function is $Q^{*}=\max_{\pi }Q^{\pi }$. The optimal greedy policy is $\pi^{*}\left(x|y\right):y\in argmax\;y^{\prime }Q^{*}\left(x,y^{\prime }\right)$ while $Q^{*}$ is obtained by Bellman’s Equation $\beta \;Q^{*}=Q^{*}$ where β is parameter of dynamic programming. The Bellman’s Equation [32] can be calculated as:

(1) $$\beta Q\left(x,y\right)=z\left(x,y\right)+d\sum_{x\in X}p\left(x^{\prime }|x,y\right)\max_{x^{\prime }\in Y}Q\left(x^{\prime },y^{\prime }\right)$$

where $z\left(x,y\right)=E_{x}^{\prime }\;z\;\left(x,y,x^{\prime }\right)$ is the expected reward.

Several agents on signalized intersections compete to achieve global network traffic objectives in traffic signal control. IA2C and MA2C equipped with memory do not rely on MDP. Here we use probability function $p(x_{t+1}|y_{1:t},x_{1:t})$ to govern state transitions. The probability function unknown to the agent takes in to account all the previous states and actions. Generally, the agent’s environment behavior is recorded in finite episodes and saved in memory for the experience. At each time step *t*, observing state $x_{t}$, agent performs an action $y_{t}$ and get a reward $z_{t}$ whose probability solely depends on $y_{t}$ and $x_{t}$. An observed history $M_{t}$ is maintained which includes all sequences from the start to the current episode such that $M_{t}=\left[x_{0},y_{0},\ldots ,s_{t-1},y_{t-1},x_{t}\right]$. In single-agent recurrent Advantage Actor-Critic neural networks the loss can be calculated as:

(2) $$\ell \left(\theta \right)=\sum_{t=0}^{t_{M}-1}\log\pi_{\theta }\left(x_{t}|h_{t}^{\pi }\right)S_{t}$$

where as the Critic loss can be calculated as:

(3) $$\ell \left(\psi \right)=\frac{1}{2}\sum_{t=0}^{t_{M}-1}{\left(Z_{t}-V_{\psi }\left(h_{t}^{V}\right)\right)}^{2}$$

In the above Equations $h_{t}^{\pi }$ and $h_{t}^{V}$ are referred to as histories that include past experiences and used to compute $\pi_{\theta }$ and $V_{\psi }$. The $Z_{t}=\overset{\wedge }{Z_{t}}+\gamma^{t_{M}-1}V_{\psi }-\left(h_{t_{M}}^{V}\right)$ is the return at *n*-steps where $n=t_{M}-t$. The sampled advantage for actions $y_{t},\ldots ,y_{t_{M-1}}$ is $S_{t}=Z_{t}-V_{\psi }-\left(h_{t}^{V}\right)$. The $\theta^{-}$ represents the actor parameter’s set while $\psi^{-}$ represents the critic parameter’s set computed at the last learning step.

In Independent Advantageous Actor Critic (IA2C) each agent *a* learned its own value function $V_{\psi_{a}}$ and policy $\pi_{\theta_{a}}$ from its experiences. Here the loss function for the actor and critic can be measured as:

(4) $$\begin{array}{c} \ell \left(\theta_{a}\right)=\sum_{t=0}^{t_{M}-1}\log\pi_{\theta_{a}}\left(y_{t,a}\;|\;h_{t}^{\pi },\nu_{a}\right)S_{t,a}+\\ \beta \sum_{y_{a\in S_{a}}}\pi_{\theta_{a}}\log\pi_{\theta_{a}}\left(y_{a}|h_{t}^{\pi },\nu_{a}\right) \end{array}$$

(5) $$\ell \left(\psi_{a}\right)=\frac{1}{2}\sum_{t=0}^{t_{M}-1}{\left(Z_{t,a}-V_{\psi_{a}}\left(h_{t}^{V},\nu_{a}\right)\right)}^{2}$$

where:

$$S_{t,a}=Z_{t,a}-V_{\psi_{a}}-\left(h_{t}^{V}\nu_{a}\right)$$

$$Z_{t,a}=\overset{\wedge }{Z_{t}}+\gamma^{t_{M}-t}V_{\psi_{a}}-\left(h_{t_{M}}^{V},\nu_{a}\right)$$

$$\overset{\wedge }{Z_{t}}=\sum_{z=t}^{t_{M}-1}\gamma^{\tau -t}\overset{\wedge }{z_{\tau }}$$

$$\overset{\wedge }{\nu_{t}}=\frac{1}{\nu }\sum_{a\in \nu }z_{t,a}$$

$$h_{t,\nu_{a}}^{\pi }=X^{\pi }\left(H_{t-\nu_{a}}\right)$$

$$h_{t,\nu_{a}}^{V}=X^{V}\left(H_{t-\nu_{a}}\right)$$

$$h_{t,\nu_{a}}=\left[\left\{x_{0,j}\right\},y_{0},\ldots .,\left\{x_{t-1,j}\right\},y_{t-1},\left\{x_{t,j}\right\}\right]with\;j\in \nu_{a}$$

In the above Equations $\theta_{a}$ and $\psi_{a}$ are the actor and critic parameters in the network. The superscript “-” shows the current values and *a* as subscript shows all the agents apart from agent “*a*”. The Algorithms 1 and 2 gives the detailed description in form of pseudo-code of the IA2C and MA2C used in our experiments.
**Algorithm 1** Independent Advantageous Actor Critic (IA2C) Algorithm for MARL approach1:Start Simulation                    ▹ SUMO is used for Simulations**Initialize**   parameters:2:$Actor\;param=\theta_{a}$ ; $Critic\;param=\psi_{a}$3:$Entropy\;loss=\pi_{\theta_{a}}$ ; $histories=h_{t}$4:$Temporal\;loss=R_{t}$5:$Sample\;advantage\;for\;action=Y_{t}$6:Policyparameter(λ)7:$State\;parameter(\bar{\nu })$**Randomize**  8:$Actor\;parameter(\theta_{a})$9:$Critic\;parameter(\psi_{a})$**Set** :10:$Entropy\;:\pi_{\theta_{a}}$≥011:$Actor\;learning\;rate:L_{\theta_{a}}$≥012:$Critic\;learning\;rate:L_{\psi_{a}}$≥013:**while** $Episodes\neq T_{total}$ **do**14:    Initialize Memory Buffer15:    **for** <Every Episode [0−n]> **do**16:        $Perform\;action\;y_{t}\;using\;current\;policy\;\lambda_{t}$17:        $Observe\;next\;state\;x_{t+1}$18:        $Store\;y_{t}\;,\;x_{t+1}\;,\;z_{t+1}\;in\;memory$**Compute** :19:        Action $y_{t}\left(x_{t\;,\;\theta_{a}^{t}}\right)\;|\;\psi_{a}$20:        Entropy $\left(h_{t}\;,\;\nu_{a}\right)=\sum_{x=0}^{k}\left(x_{t}.k\right)∵k\in \psi_{a}$21:        Using $y_{t},z_{t},x_{t+1}$**Get**  22:        $Actor-loss:\ell (\theta_{a})$23:        $Critic-loss:\ell (\psi_{a})$24:        $TD-Error:R_{t},a$**Update**  25:        $Actor\;param=\theta_{a}$26:        $Critic\;param=\psi_{a}$27:    **end for**28:**end while**

Because all actions have equal probabilities at the start of training, the entropy loss of policy $\pi_{\theta_{a}}$ has been included in Equation (4), adjusted by the parameter β, to allow for rapid exploration. If the observation of agent *a* is restricted to $\nu_{a}$, it will affect both $V_{\psi_{a}}$ and $\pi_{\theta_{a}}$ through $H_{t}\;,V_{a}$. It suffers from partial observability at one side, but it also allows the values of $V_{a}$ and $\pi_{\theta_{a}}$ to converge towards optimum.

MA2C stabilizes the learning process and weak convergence by communicating with neighboring agents. A spatial discount factor weakens the reward and state signals of other agents not present in ${ℜ}_{a}$ by excluding them for reward computation. So Equations (4) and (5) become:

(6) $$\begin{array}{cc} \ell (\theta_{a}) & =\sum_{t=0}^{t_{M}-1}\log\pi_{\theta_{a}}\left(y_{t,a}{\tilde{h}}_{t}^{\pi },\nu_{a},\pi_{t-1},{ℜ}_{a}\right){\tilde{S}}_{t,a}\\ & +\beta \sum_{y_{a\in S_{a}}}\pi_{\theta_{a}}\log\pi_{\theta_{a}}\left(y_{a}{\tilde{h}}_{t}^{\pi },\nu_{a},\pi_{t-1},{ℜ}_{a}\right) \end{array}$$

(7) $$\ell \left(\psi_{a}\right)=\frac{1}{2}\sum_{t=0}^{t_{M}-1}{\left({\tilde{Z}}_{t,a}-V_{\psi_{a}}\left({\tilde{h}}_{t}^{V},\nu_{a}\;,\;\pi_{t-1},{ℜ}_{a}\right)\right)}^{2}$$

**Algorithm 2** Multi-Agent Advantageous Actor Critic (MA2C) Algorithm for MARL approach1:Start Simulation                             ▹ SUMO is used for Simulations**Initialize**   parameters:2:$\alpha_{a},\beta_{a},\gamma_{a},T,\eta_{\psi_{a}},\eta_{\theta_{a}},t,j$3:Memory Buffer* [M]*4:$Initial\;state\;x_{0}$5:policyparameterλ−1**Set**  6:t=0,j=07:**while** $Episodes\neq T_{total}$ **do**                          ▹ Until Simulation Ends8:    **for** < a∈ν> **do**9:        Calculate sample local policy10:        $\lambda_{t,a}=f_{\theta_{a}}\;\left(h_{a}\;,\;\lambda_{t-1}\right)$11:        Sample $\nu_{t,a}\;from\;\lambda_{t,a}$12:        Collect13:        $z_{t,a}=\frac{1}{\nu_{a}}\left(\sum \alpha \;z_{t.j}\right)+z_{t,a}$14:        $x_{t,a}=x_{\nu_{a,t}}U\lambda N_{a,t-1}$**Update**  15:        A2C16:        Memory buffer*|M|*17:        Perform Action18:    **end for**19:    **end while**20:    **while** $Episodes==T_{total}$ **do**21:        Re-initialize $state\left(x_{0}\right),policy\left(\lambda -1\right),t\leftarrow 0$22:        **if** J=|M| **then**23:           **for** < a∈ν> **do**24:               Calculate:25:               $Z_{t,a}\;,\;\forall \tau \in |M|$26:               $Y_{t,a}\;,\;\forall \tau \in |M|$27:               $Actor-Loss=\ell (\theta_{a})$28:               $Critic-Loss=\ell (\psi_{a})$**Update**  29:               Policy parameters $\left(\psi_{a},\theta_{a}\right)$ with $\eta_{\psi }▽\ell \left(\psi_{a}\right)$ and $\eta_{\theta }▽\ell \left(\theta_{a}\right)$30:           **end for**31:        **end if**32:    **end while**

We can see in the above equation that the learning process is more stable because of the fingerprints $\pi_{t-1},{ℜ}_{a}$. These are the inputs to the $V_{\psi_{a}}$ and secondly there is more correlation between the local region observations ${\tilde{x}}_{t},\nu_{a},\pi_{t-1},{ℜ}_{a}$ and spatially discounted return ${\tilde{Z}}_{t,a}$. In this way, agents take the current state of the intersection and considering the waiting time and queue lengths of the vehicles turn the appropriate signal on and off. It also communicates with the neighboring intersections for information sharing so that more appropriate actions could be taken by considering the neighboring intersections also. The Algorithms 1 and 2 shows the description of IA2C and MA2C.

In the second phase of our approach, we are using a routing strategy to manage the flow of traffic. Due to high traffic volumes, the congestion behind the intersections also increases and to avoid traffic jams and long delays we rerouted the vehicles on other paths toward their destination in a reliable and cost-efficient manner. The information about the alternate routes and expected travel time on the routes should be considered appropriate to ensure cost-efficient routing. At first, we used the Dijkstra algorithm [33] to compute the shortest paths between the origin and destinations. We then computed all the alternate paths and stored them in the route file. This pre-computed information is used to re-route vehicles efficiently. During congested hours, while our intelligent traffic signal is managing traffic at intersections the vehicles that are not yet reached the intersection are considered the current state of the intersection. If the total travel time of the current path and wait time on the intersection is more than the travel time of the second alternate path then the vehicle will be rerouted to the alternate path but if the time is less then the vehicle will stay on the same route and wait. In this way vehicles at the intersection and behind the intersections are load-balanced and traffic flow is improved by selecting better paths. In Algorithm 3, a detailed description is given of the process. We used different detectors, re-routers, and sensors for this phase. In SUMO there are many features available for the routing of vehicles at simulation time. We used re-routers and edge weights and multiple TraCI functions for route assignments for example using the methods “traci.vehicle.reroute Travel time” or “traci.vehicle.change Target”. Routes can be computed using “traci.simulation.find Route” and applied using “traci.vehicle.setRoute”.
**Algorithm 3** Routing of vehicles to alternate paths1:Start Simulation                               ▹ SUMO is used for Simulations**Initialize**  2:Memory**Set**  3:$d_{c}=critical\;density\;;\;d_{j}=jam\;density$4:Generate vehicles with unique ID [v1,v2,…,Vn]5:Get O-D matrix of each vehicle**Compute**  6:Shortest path ‘$S_{p}$’ with travel time ‘$X_{t}$’7:All alternate paths $\left[A_{p}=A_{p_{1}},A_{p_{2}},\ldots ,A_{p_{n}}\right]$ with their travel times8:**for** <Vehicles at Intersection I> **do**9:    Get the current state of Intersection10:    **while** $d\neq d_{c}$ **do**                              ▹ When density is not critical11:        wait on the same route12:    **end while**13:    **if** $d\to d_{c}$ **then**14:        Compute wait time ‘$W_{t}$’ at intersection15:        Add `$W_{t}$’ to ‘$X_{t}$’ of current route16:        Get Updated time ‘$U_{t}$’ such as $U_{t}=W_{t}+X_{t}$17:    **end if**18:    **while** $d==d_{j}$ **do**                              ▹ When traffic jam occurs19:        **if** $U_{t}S_{p}>A_{p_{1}}$ **then**    ▹ When wait time on shortest path is greater then alternate path’s travel time20:           Reroute the vehicles to alternate path21:        **end if**22:    **end while**23:**end for**

## 4. Experimental Setup

This section gives an overview of the proposed approach’s system implementation and experimental details.

### 4.1. Simulator Used for Experiments

Simulation plays a vital role in solving real-world problems efficiently and safely. It is an essential analytical technique that is easy to test, communicate, and understand. Simulation provides valuable solutions across industries and disciplines by clearly understanding complex systems. In traffic control systems, traffic simulations provide a critical way to understand the traffic dynamics and control operations, resulting in the development of better ideas, new algorithms, and efficient systems [34].

In this paper, we used SUMO (“Simulation of Urban MObility”) [35], which is an open-source, microscopic traffic simulator. It is used for importing/creating road networks, generating routes, generating different vehicles, assigning different traffic densities to the roads, and embedding other infrastructural systems on the roads for the achievement of a complex and modern road network. The external applications provide a socket-based interface through which different algorithms could be implemented and tested in different scenarios. The simulation parameters are given in Table 1.

### 4.2. Road Network Description

**Figure 2:**
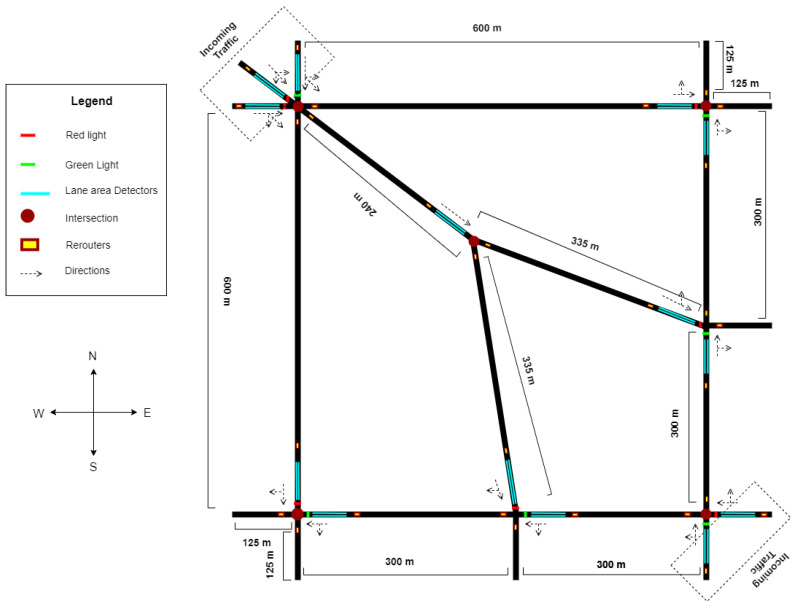
Road Network used for experiments with seven intersections.

In modern cities, developing methods to access the functioning of a road network is one of the main challenges [36]. Traffic flow models are developed for specific situations for observing traffic flow and managing traffic. We used the road network shown in Figure 2 for our simulations. The road network consists of roads of various lengths and intersections. We used seven intersections with our proposed intelligent traffic lights at each intersection. There are varying roads between the intersection and many alternate routes for reaching from origin to destination. We also used detectors and re-routers of each road segment to route vehicles to alternate paths. The red line in Figure shows that the traffic signal is closed, and the green shows that signal is on. The cyan color line shows the detectors, and the yellow rectangle with a red outline shows the re-routers embedded in the positions on roads. Each road consists of 8 lanes i.e., four incoming and four outgoing. This road network is inspired by the road network used in [37,38] consisting of different segments with several intersections and configurations. The design of the road network is mirrored version of the road network used in above mention articles with varying road lengths and detector spans. Different complexities of real road networks have been incorporated into this road network with different segments with several intersections and configurations. In previous chapters, we used single intersections for our experiments therefore road network is based on single intersections. In this chapter, our experiments are based on Multi-Agent Reinforcement Learning therefore the road network is based on multiple intersections.

### 4.3. Traffic Assignment in the Road Network

By traffic assignment, we typically refer to the number of vehicles present on the road network. Different types of models such as Traffic Flow can be used for this purpose. In the traffic assignment problem, the density of vehicles is considered as between each O-D (Origin-Destination) matrix. We used 7200 vehicles for our simulations on the road of lengths of 600/300 m. Most vehicles pass through intersections and are controlled by intelligent traffic lights using deep reinforcement learning. This is improving congested situations while making the traffic flow more efficient some vehicles are rerouted to alternate paths.

### 4.4. Detectors and Re-Routers

There are several traffic data collection methods in AVs. The first technology is “in situ” and the second is “in-vehicle”. The “in situ” technology consists of the data collected from detectors embedded on the roadsides. There are generally two categories of traffic count strategies: the intrusive, which uses sensors or data recorders placed on different road segments, and the non-intrusive, which consists of data obtained from remote observations. Similarly, re-routers are used to change the vehicle’s route when vehicles enter the specified edge.

In these experiments, we used 13 detectors and 29 re-routers placed in specific locations to obtain the number of vehicles that entered the road segment. The re-routers are used to reroute the vehicles to alternate paths when the number of vehicles increases to the specific limit.

### 4.5. Intelligent Traffic Signal

In SUMO, fixed traffic lights are generated with 90 s cycle time by default pre-time. The cycle time could be changed according to the requirement. Between the main phases, green time is distributed equally and is followed by the yellow phase. The yellow phase is dependent on the max speed of roads and could be changed according to requirements.

In this paper, we used seven traffic lights on seven intersections. The green duration is 4 s, the yellow duration is 2 s, and the red is dependent on the number of vehicles at the intersection. The duration of these phases is dependent on the multiple characteristics of traffic such as the number of vehicles, available space on road, number of vehicles in the queue, etc. [39]. The green phase duration can be restricted to a min/max limit or could be allowed to any value according to the requirement [40]. Reinforcement learning is used to make these traffic signals intelligent to work according to the current situation at the intersection. Using MARL, these traffic signals can also communicate with neighboring signals and get the traffic condition on those signals to make traffic decisions accordingly.

### 4.6. Neural Network Details

We designed a model that deals with spatiotemporal data, which means that we not only need to collect and process data at a particular time; instead, we need to get information about both time and location. Utilizing a spatiotemporal approach, both of the Multi-Agent Reinforcement Learning techniques, including MA2C and IA2C that we implemented, use an Artificial Recurrent Neural Network (A-RNN). Our A-RNN model consists of 7 layers, including the input and the output layers. Other than the input and the output layer, the other five hidden layers consist of 2 convolutional layers, 2 Long Short-Term Memory (LSTM) layers, and a single fully connected layer. The input layer gathers the data directly from the simulation and divides them into batches, including wave, wait, and neighboring policies (only for MA2C) before sending them to the fully connected layers that we used for processing. These fully connected layers contain 128, 32, and 64 neurons for each input data type (wait, wave, neighboring) received. In our approach, an LSTM layer takes a combined input from the fully connected layers for making predictions using previous historical data it receives during training and sends the results of the predictions to the output layer. We used Rectifier Linear Unit (ReLu) activation function in the output layer for the actor and the critic in both of the algorithms implemented. We also set the hidden size for both the actor and the critic to 32, along with the learning rate of 0.01. Other than that, we also used the RMSprop optimization function to increase the learning rate of the neural network and the mean squared error function for computing loss.

We trained our designed Artificial Recurrent Neural Network (A-RNN) at 200 epochs and a gamma value of 0.99. The gamma value itself is responsible for setting the impact of the gradient on weights during model training. Our training network follows a centralized learning policy for both MA2C and IA2C algorithms. By a centralized learning policy, we mean that the training or critic is kept centralized, and the execution or actor is kept decentralized. A centralized agent can collect the data from all neighboring agents located in the MARL network, whereas a decentralized agent can only collect its data and compute rewards accordingly. In terms of replay buffer, we set the value at 10,000, with network parameters updated every 100 steps constantly. We set the GUI parameter of the model training to TRUE so that the simulations run automatically before every episode. At last, we also used a common technique known as an epsilon-greedy policy to decide whether to perform exploration or exploitation. For the epsilon greedy policy, we set the parameters to start from 0.9 and end at 0.01 with a decay rate of 200, and the decision between exploitation and exploration is made randomly. Training parameters are given in Table 2.

## 5. Results and Discussions

In this section, we will present the results of our experiments and discuss them in detail. Traffic signals based on MARL are evaluated in a sumo-simulated environment. Our road network consists of seven intersections with variant traffic flows. The objective of this type of network was to get a realistic and challenging environment for experiments. Different types of experiments are used to demonstrate the robustness and efficiency of our proposed approach. First, we used pre-timed fixed signals in our road network and observed the congestion at different intersections. Then we used IA2C to train our traffic signals and observe the traffic conditions, and then we applied MA2C to observe the traffic conditions, and finally, we used rerouting with MA2C to observe the current state of the intersection.

**Figure 3:**
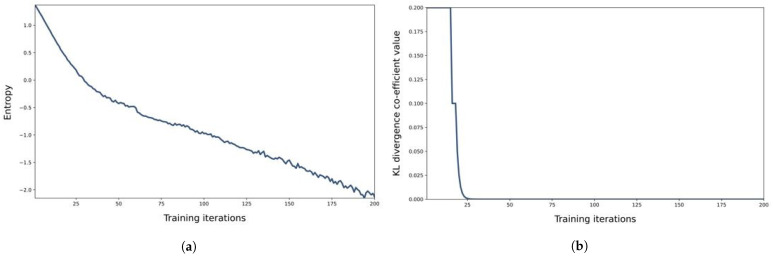

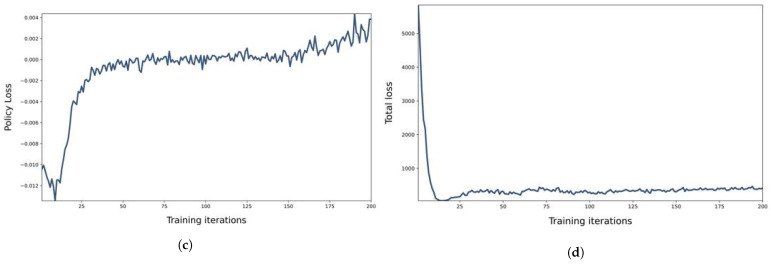
Results of Entropy, KL Divergence, Policy Loss and Total loss. (**a**) High to Low Entropy distribution representation. (**b**) KL Divergence. (**c**) Policy Loss. (**d**) Total Loss.

In Figure 3 we can see the entropy regularization in RL algorithms. Entropy is the predictability of an agent’s action, which will result in the highest reward. We can say that entropy is related to the certainty of its policy: entropy is high when certainty is low, and entropy is low when certainty is high. In Figure 3a, we can see that at the start of the simulation, entropy is high, which means there is less certainty about the actions, which gives us high rewards, but as the agent starts learning about the environment, the entropy becomes low, and we got the optimal policy. When the agent obtains a positive reward by an action, it is always used by the agent in the future because it knows that this action gives a positive reward. However, another type of action gives a much higher reward than the previous action, but the agent never uses it in the future because it knows that this action only exploits what the agent already learned. Such actions stop agents from exploring other actions and cause agents to get stuck in the local optimum. To obtain global optimum, entropy is used that encourages exploration which is very important for sparse rewards where feedback of actions is not regular and causes overestimation of some rewards and repetition of actions. Exploration results in fine-tuning policies, and agents become more robust to rare and abnormal events. In Figure 3b the Kullback–Leibler (KL) divergence is shown for our experiments. It measures the difference between two probability distributions: true distribution and approximate distribution. KL-divergence measures the distance between the approximate distribution to the true distribution. In the figure, we can see how kl-divergence decreases from maximum to minimum as the agent learns the policy. In Figure 3c,d, we can observe the policy loss and total loss in our system. The policy network predicts the probability distribution of actions for optimal policy after training. The data for training is obtained by interacting with the environment from experience and storing results as an episode of experience. So we can say that the training process depends on many epochs, and in each epoch, there are two stages: in the first stage, it obtains training data for an epoch from the environment, and in the second stage, it trains the policy network using training data. When the policy network gets the training data at the end of the episode, it takes action from sampled data for the current state and predicts the action probability. The reward from data samples is used to calculate the discounted return, and loss to train is computed by action probability as shown in Figure 3c,d. The policy network learns with each passing iteration, predicts better actions, and finally finds an optimal policy.

**Figure 4:**
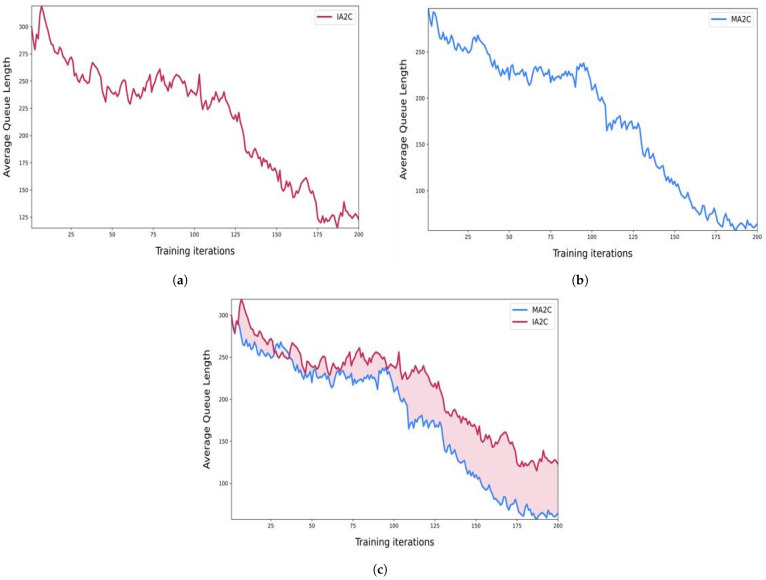
Average Queue Length comparisons. (**a**) Average Queue Length IA2C. (**b**) Average Queue Length MA2C. (**c**) Comparisons for Average Queue Length IA2C and MA2C.

**Figure 5:**
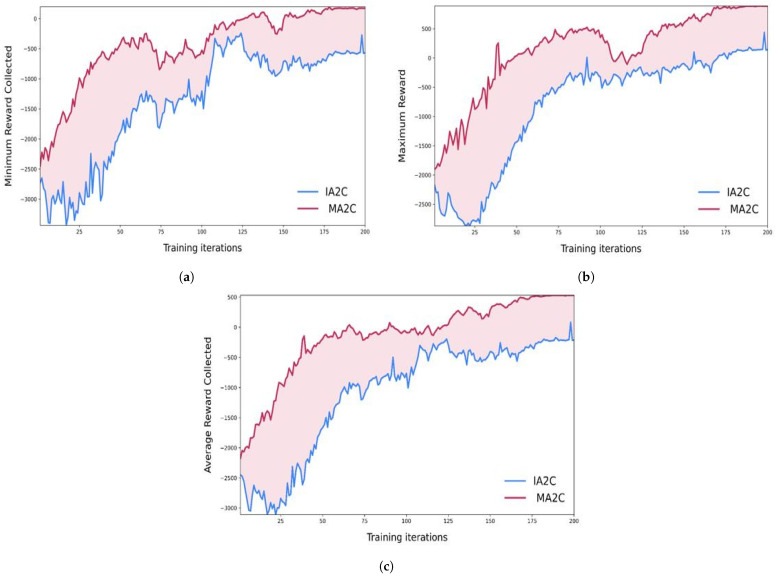
Results of reward value comparisons. (**a**) Minimum Reward Comparison for IA2C and MA2C. (**b**) Maximum Reward Comparison for IA2C and MA2C. (**c**) Average Reward Comparison for IA2C and MA2C.

**Figure 6:**
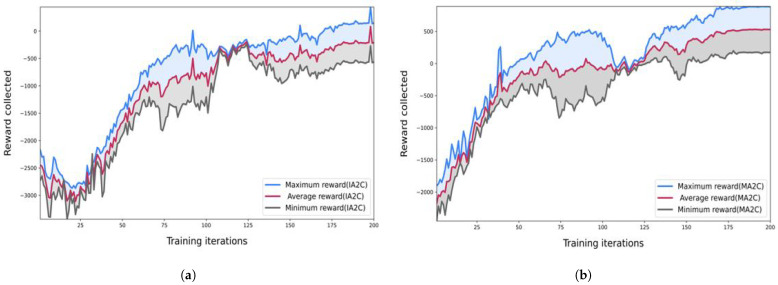
Results of maximum reward comparisons for IA2C and MA2C. (**a**) Maximum reward comparisons for IA2C. (**b**) Maximum Reward Comparison MA2C.

In Figure 4 we can see that the queue lengths in our road network started minimizing as we implemented the reinforcement learning algorithms to our traffic signals. Figure 4a showed the results when we implemented traffic signals with the IA2C algorithm. Figure 4b showed the queue length when we used MA2C on traffic signals. Finally, Figure 4c shows the comparison between IA2C and MA2C. Figure 5 shows the reward comparisons for IA2C and MA2C. Figure 5a shows the minimum rewards of IA2C and MA2C as well as their comparisons. Similarly, Figure 5b,c shows the minimum and average rewards and their comparisons. We can see in Figure 5 that MA2C is performing better in our scenarios than IA2C. The reward function in RL is crucial, and it is very challenging to find an appropriate reward function as it determines the system’s behavior. In Figure 5, we explore the impact of different parameters on the performance of our reward function. We find that MARL has the most suitable variables for our design function. In Figure 6 we did the comparisons of minimum, average and maximum rewards for IA2C and MA2C. Figure 6a shows the minimum, maximum, and average reward comparisons for IA2C, and Figure 6b shows the minimum, maximum, and average reward comparisons for MA2C. We can see that the maximum reward is improving with each iteration for both IA2C and MA2C.

**Figure 7:**
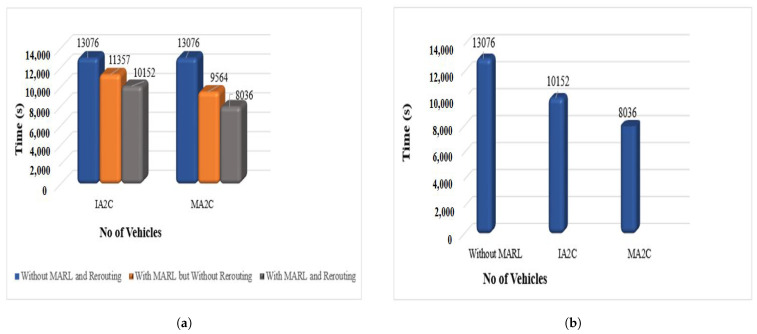
Comparisonresults of improved performance by our proposed approach. (**a**) Comparison with and without implementing MARL and Rerouting. (**b**) Comparison with and without implementing MARL.

Figure 7 shows the overall improvement in performance by our proposed approach. In Figure 7a the improvement in performance is shown by the implementation of a rerouting strategy to our experiments. In the blue bar of the figure, we can see that all vehicles are taking almost 13,076 (s) when there are pre-timed fixed signals used, and no MARL and rerouting are implemented in our experiments. When we implemented MARL with IA2C and MA2C, the performance improved from 13,076 (s) to 11,375 (s) in IA2C and 9564 (s) in MA2C, respectively, as shown in an orange bar in Figure 7a. The rerouting further improved the performance by 10,152 (s) in IA2C and 8036 (s) in MA2C, as shown in the grey bar of the figure. If we further go into the details of performance, we can see that in Figure 7b three bars represent the performance of traffic lights without implementing the proposed approach and improvement in performance with IA2C and then further improvement by MA2C with rerouting. The first bar shows that all vehicles take almost 13,076 (s) to pass through intersections when there are pre-timed fixed signals. The second bar shows the implementation of MARL with the IA2C algorithm, and all the vehicles are taking 10,152 (s) to reach their destination, which means there is an improvement of almost 17% from pre-timed signals. The third bar of Figure 7b shows that when we implemented MARL with MA2C, all vehicles took 8036 (s) to reach their destination, which means it improved the performance by almost 26%. The incremental improvement of performance from pre-timed fix signals is almost 38%.

**Figure 8:**
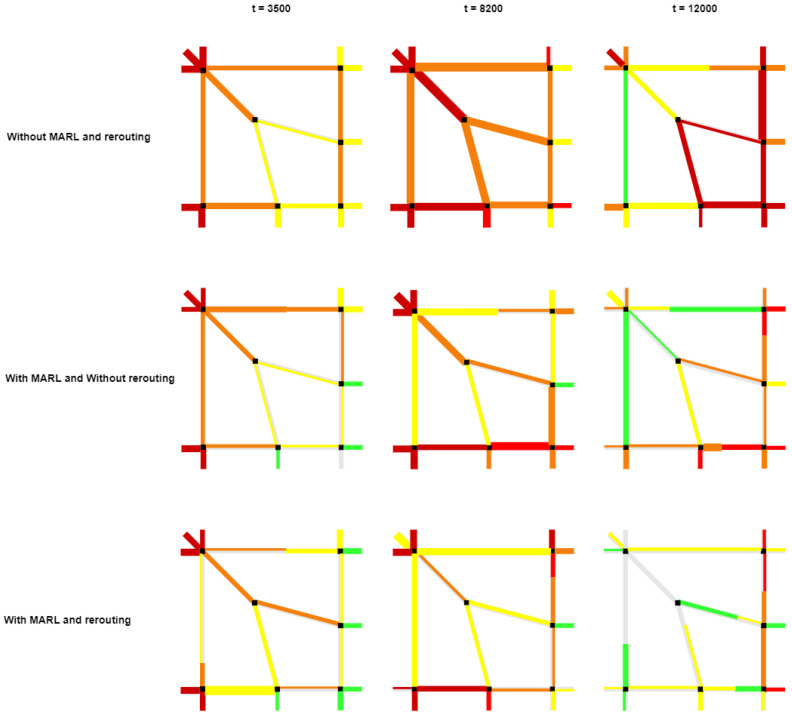
Congestion Model for improved traffic flow by our proposed approach.

Finally, in Figure 8 we can see the improvement in traffic by the removal of congestion on our road network model. The traffic condition along each row is visualized as a line segment. The colors are indicating the queue lengths or congestion level. The grey color indicates the 0% traffic, the green color 25%, the yellow 50%, the orange 70%, and the red color indicates 90% traffic. Intermediate traffic condition is shown as interpolated color and thickness of the line indicates the intersection delay (the thicker line the more waiting time at the intersection). The top three images in Figure 8 show the congestion situation with red lines on the roads when we used pre-timed fixed signals. Then the middle three images show the traffic situation on the roads when we used Multi-Agent-based reinforcement learning algorithms in our traffic lights. We can see that it reduced the congestion to many extents and improved traffic flow at intersections. Finally, when we used MARL and rerouting to manage the traffic congestion, it improved the traffic condition and cleared most of the congested areas, as shown in the last three images of Figure 8.

A combination of techniques is applied in this presented work which yields promising results in a Multi-Agent context. Multiple agents in Multi-Agent scenarios are trained to control traffic signals to minimize congestion and improve the traffic flow. The agents interact with the external environment and with each other by sharing information and obtaining the optimum behavior as shown above. However, as the number of agents increases, total information sharing and full observation become limiting factors. In both IA2C and MA2C, agents operate on local observation but differ in the reward calculation method. In IA2C reward is measured globally and agents are not allowed to communicate while in MA2C reward is measured locally, allowing agents to communicate and share information. We can see in the above-presented results that MA2C outperforms IA2C because interacting with neighboring agents helps modification of the behavior of the agent according to the real-time situation. This continuously changing behavior can cause stability issues and yield unsteady dynamics where agents continuously attempt to adapt the variations without converging to a steady state.

## 6. Conclusions

This paper proposed an approach where we used Multi-Agent Reinforcement Learning to make traffic signals intelligent and rerouting to further improve the traffic conditions on the roads. We used two algorithms, IA2C and MA2C, for adaptive traffic signals. At the intersections, intelligent traffic signals control the traffic by looking at the current state of the intersection. The traffic that is not yet reached the intersection is informed about the congestion at the intersection and then rerouted to alternate paths toward their destination. Results showed an improvement in results of almost 38%. Multi-Agent reinforcement learning is a very effective approach in traffic light control but one of the major challenges it faces is “curse of dimensionality”. As the number of agents grows, the state-action space grows exponentially. Scalability can be achieved by the paradigm of centralized training with decentralized execution. Another challenge in a Multi-Agent environment is that the objectives of the agents do not need to be aligned which could affect the efficiency and robustness of the system. As future work, The proposed approach in this paper could be extended to more complex road networks with multiple signal intersections and real-world road data sets, including more vehicles’ coordination with each other and with infrastructure. This could lead us to better traffic flow management to help support the concept of futuristic, intelligent cities.

## Figures and Tables

**Table 1:** Simulation Parameters.

Parameter	Value
Transmission range for incoming traffic	125 (m)
Transmission range between intersections	600 (m)/300 (m)
Transmission range for alternate paths	240 (m)/335 (m)
Number of Vehicles per Episode	7200
Simulation Time	13,076
Number of Intersections	07
Number of traffic lights	7
Number of lane area detectors	14
Distance between lane area detectors	75 (m)
Number of re-routers	29
Green duration	4 (s)
Yellow duration	2 (s)
Simulator	SUMO

**Table 2:** Training Parameters.

Parameter	Value
GUI	TRUE
Number of episodes	200
Batch size	100
Max steps	None
Reward scale	1.
Memory size	10,000
Loss function	MSE (Mean squared error)
Training strategy	Centralized
Total NN Layers	7
Fully Connected Layer	1
Convolutional Layers	2
LSTM layers	2
Activation function	ReLU (Rectifier Linear Unit)
Gamma value	0.99
FC wave	128
FC wait	32
lstm	64
Optimization function	rmsprop
Actor hidden size	32
Critic hidden size	32
Actor learning rate	0.01
Critic learning rate	0.01
Epsilon start	0.9
Epsilon end	0.01
Epsilon decay	200

## Data Availability

Not applicable.
